# Microcirculatory perfusion disturbances following cardiac surgery with cardiopulmonary bypass are associated with in vitro endothelial hyperpermeability and increased angiopoietin-2 levels

**DOI:** 10.1186/s13054-019-2418-5

**Published:** 2019-04-11

**Authors:** Nicole A. M. Dekker, Anoek L. I. van Leeuwen, Willem W. J. van Strien, Jisca Majolée, Robert Szulcek, Alexander B. A. Vonk, Peter L. Hordijk, Christa Boer, Charissa E. van den Brom

**Affiliations:** 1 Amsterdam UMC, Vrije Universiteit Amsterdam, Anesthesiology, Amsterdam Cardiovascular Sciences, Amsterdam, The Netherlands; 2 Amsterdam UMC, Vrije Universiteit Amsterdam, Physiology, Experimental Laboratory for Vital Signs, Amsterdam Cardiovascular Sciences, Amsterdam, The Netherlands; 3 Amsterdam UMC, Vrije Universiteit Amsterdam, Cardiothoracic Surgery, Amsterdam Cardiovascular Sciences, Amsterdam, The Netherlands; 4 Amsterdam UMC, Vrije Universiteit Amsterdam, Pulmonology, Amsterdam Cardiovascular Sciences, Amsterdam, The Netherlands

**Keywords:** Cardiopulmonary bypass, Angiopoietin-2, Capillary permeability, Microcirculation, Endothelium

## Abstract

**Background:**

Endothelial hyperpermeability following cardiopulmonary bypass (CPB) contributes to microcirculatory perfusion disturbances and postoperative complications after cardiac surgery. We investigated the postoperative course of renal and pulmonary endothelial barrier function and the association with microcirculatory perfusion and angiopoietin-2 levels in patients after CPB.

**Methods:**

Clinical data, sublingual microcirculatory data, and plasma samples were collected from patients undergoing coronary artery bypass graft surgery with CPB (*n* = 17) before and at several time points up to 72 h after CPB. Renal and pulmonary microvascular endothelial cells were incubated with patient plasma, and in vitro endothelial barrier function was assessed using electric cell–substrate impedance sensing. Plasma levels of angiopoietin-1,-2, and soluble Tie2 were measured, and the association with in vitro endothelial barrier function and in vivo microcirculatory perfusion was determined.

**Results:**

A plasma-induced reduction of renal and pulmonary endothelial barrier function was observed in all samples taken within the first three postoperative days (*P* < 0.001 for all time points vs. pre-CPB). Angiopoietin-2 and soluble Tie2 levels increased within 72 h after CPB (5.7 ± 4.4 vs. 1.7 ± 0.4 ng/ml, *P* < 0.0001; 16.3 ± 4.7 vs. 11.9 ± 1.9 ng/ml, *P* = 0.018, vs. pre-CPB), whereas angiopoietin-1 remained stable. Interestingly, reduced in vitro renal and pulmonary endothelial barrier moderately correlated with reduced in vivo microcirculatory perfusion after CPB (*r* = 0.47, *P* = 0.005; *r* = 0.79, *P* < 0.001). In addition, increased angiopoietin-2 levels moderately correlated with reduced in vitro renal and pulmonary endothelial barrier (*r* = − 0.46, *P* < 0.001; *r* = − 0.40, *P* = 0.005) and reduced in vivo microcirculatory perfusion (*r* = − 0.43, *P* = 0.01; *r* = − 0.41, *P* = 0.03).

**Conclusions:**

CPB is associated with an impairment of in vitro endothelial barrier function that continues in the first postoperative days and correlates with reduced postoperative microcirculatory perfusion and increased circulating angiopoietin-2 levels. These results suggest that angiopoietin-2 is a biomarker for postoperative endothelial hyperpermeability, which may contribute to delayed recovery of microcirculatory perfusion after CPB.

**Trial registration:**

NTR4212.

**Electronic supplementary material:**

The online version of this article (10.1186/s13054-019-2418-5) contains supplementary material, which is available to authorized users.

## Background

Cardiac surgery with cardiopulmonary bypass (CPB) is often complicated by tissue edema as a consequence of a systemic inflammatory response and vascular endothelial hyperpermeability [1–3]. We previously showed that this impairment of endothelial barrier function and subsequent fluid shift hampers microcirculatory perfusion [4–6] and contributes to the development of postoperative organ dysfunction, in particular acute kidney and lung injury [7].

The angiopoietin/Tie2 system has been proposed as a key signaling pathway in CPB-related endothelial hyperpermeability [8–11]. Tie2 is a vascular restricted tyrosine kinase receptor with specificity for angiopoietin-1 and angiopoietin-2 binding [12]. In quiescence, angiopoietin-1 binds to Tie2, resulting in receptor phosphorylation and inhibition of inflammation. During stress as observed in CPB, stored angiopoietin-2 is released from Weibel-Palade bodies and competes with angiopoietin-1 for Tie2 binding which antagonistically reduces endothelial barrier function and increases inflammation [12].

The potential of angiopoietin-2 as a biomarker for endothelial dysfunction and unfavorable outcome has been extensively investigated in septic populations [13–15], but has been restricted to in vitro models [3, 8] or the evaluation of plasma markers in cardiac surgery patients [9, 10, 16]. Increased plasma angiopoietin-2 levels following CPB are associated with prolonged mechanical ventilation [8] and acute kidney injury [16]. We and others showed that the onset of CPB is associated with an acute impairment of in vitro endothelial barrier function [3, 8]. In addition, we previously showed that targeting Tie2 with an angiopoietin-1 mimetic could reduce pulmonary vascular leakage and preserve in vivo microcirculatory perfusion during and after CPB in an experimental model [5], implying the importance of angiopoietin-1-dependent Tie2 signaling and endothelial integrity to maintain microcirculatory perfusion and organ function after CPB.

Although recent studies emphasized the biological and clinical relevance of increased angiopoietin-2 levels in the first hours following CPB [8–10], the connection between postoperative angiopoietin-2 levels, endothelial barrier function, and microcirculatory perfusion following CPB remains to be elucidated. We therefore aimed to investigate the postoperative effects of cardiac surgery with CPB on in vitro renal and pulmonary endothelial barrier function and their relation with circulating angiopoietin/Tie2 and microcirculatory perfusion.

## Methods

### Study design

The *GlyCar* study was approved by the Human Subjects Committee of the Amsterdam UMC (13.291, NTR4212, Amsterdam, The Netherlands), and clinical data were previously published [6]. Patients (age 18–85 years) scheduled for elective coronary artery bypass graft (CABG) surgery with cardiopulmonary bypass (CPB) were included. Exclusion criteria were re-operation, emergency operation, patients with type 1 diabetes mellitus, a body mass index over 35 kg/m^2^, and patients with a history of hematologic, hepatic, or renal diseases (eGFR < 50 ml/min).

Patients underwent standard anesthesia and cardiopulmonary bypass protocols as described previously [6]. Briefly, anesthesia was induced using intravenous sufentanil (1–3 μg/kg), combined with rocuronium (0.5–1.0 mg/kg) and midazolam (0.1 mg/kg), and maintained by continuous propofol infusion (100–400 mg/h). The extracorporeal circuit consisted of a centrifugal blood pump and a heatercooler device (Stockert Instrumente GMBH, Munich, Germany), a phosphorylcholine-coated tubing system (P.h.i.s.i.o., The Sorin Group, Mirandola, Italy), and a hollow fiber oxygenator (Affinity, Medtronic, Minneapolis, MN, USA). CPB was initiated after heparin administration (300 IU/kg) when target activated clotting time (ACT) exceeded 480 s, and supplementary doses were administered if necessary. CPB flow was maintained at 1.8–2.6 l/min/m^2^ with mild hypothermia (34–36 °C). At the end of surgery, anticoagulation with heparin was reversed using protamine in a 1:1 ratio to achieve normal ACT. A cell saver (Autolog, Medtronic, Minneapolis, USA) was used for autologous red blood cell transfusion [17].

### Collection of blood samples

Arterial blood was collected after induction of anesthesia before onset of CPB (pre-CPB), after initiation of CPB (CPB), 1 h after weaning from CPB (post-CPB), and 24 h and 72 h following surgery (+ 24 h and +  72 h, respectively) and immediately centrifuged at 4.000 G for 10 min at 4 °C. Plasma supernatant was centrifuged for another 5 min at 12.000*g* at 4 °C to obtain platelet-free plasma. Platelet-free plasma was snap frozen in liquid nitrogen and stored at − 80 °C. Plasma concentrations of angiopoietin-1 (DANG10), angiopoietin-2 (DANG20), and soluble Tie2 (DTE200, R&D Systems, Biotechne, Minneapolis, MN, USA) were measured using commercially available enzyme-linked immunosorbent assays in accordance with the manufacturer’s instructions.

### Cell culture

Human primary glomerular endothelial cells isolated from human glomerular tissue were obtained from three healthy donors obtained from Cell biologics (H-6014G, Cell Biologics Company, Chicago, USA) and pooled and cultured on gelatin-coated T25 flasks in complete medium in an atmosphere of 95% air and 5% CO_2_ at 37 °C (Additional file 1: Supplemental methods). Human pulmonary microvascular endothelial cells were isolated from healthy lung tissue obtained from three donors during lobectomy (Amsterdam UMC – location VU University Medical Center, Amsterdam, The Netherlands) and cultured as described previously [18].

### Endothelial barrier function

Electric Cell-substrate Impedance Sensing (ECIS, Applied BioPhysics, Troy, NY, USA) was used to measure impedance of endothelial cells [19]. Confluent glomerular endothelial cells or pulmonary microvascular endothelial cells were incubated for 1 h with 1% human serum albumin (HSA) in bare medium followed by the addition of 10% platelet-free plasma obtained from cardiac surgery patients at different time points before and after CPB as described above (Additional file 1: Supplemental methods). Resistance of endothelial monolayers was continuously measured at 4.000 Hz for 3 h until steady state was reached using ECIS software (v1.2.210.0 PC; Applied Bio-Physics). Measurements were performed in duplicate, and data were normalized to baseline.

### Immunofluorescence staining

Immunofluorescence was used to visualize endothelial cell structures after exposure to plasma obtained from patients either before (*n* = 6) or after (*n* = 6) exposure to CPB. Glomerular endothelial cells or pulmonary microvascular endothelial cells were exposed to plasma for 3 h. Subsequently, endothelial cells were stained for VE-cadherin (SC-6458, Santa Cruz, Dallas, TX, USA) and actin (acti-stain Phalloidin670, Cytoskeleton, Denver, CO, USA). Nuclei were stained using DAPI (1:500; Thermo Fisher Scientific, Waltham, MA, USA) (Additional file 1: Supplemental methods).

### Microcirculatory perfusion

Microcirculatory perfusion of all patients in this study, represented as percentage of perfused vessels (PPV, %), has previously been reported [6]. Briefly, sublingual microcirculatory perfusion was measured using non-invasive side stream dark field (SDF) video microscopy (Capiscope HVCS-HR, KK Technology, Honiton, UK) to visualize flowing erythrocytes based on the absorbance spectrum of hemoglobin. Videos of around 10 s were obtained in three different sublingual areas per time point. Videos were analyzed off-line using automatic vascular analysis software (AVA 3.0, Microvision Medical, Amsterdam, The Netherlands) according to microvascular scoring recommendations by De Backer et al. [20]. Vessels were manually identified and scored for flow. Micro-vessels (diameter ranging from 5 to 25 μm) scored with absent or intermittent flow (at least 50% of the time absent flow) were classified as non-perfused, and micro-vessels scored with continuous flow were classified as perfused vessels. Subsequently, the proportion of perfused vessels (PPV; in %) was automatically calculated as the proportion of perfused micro-vessels from the total amount of identified micro-vessels.

### Statistical analysis

Data were analyzed with GraphPad Prism 7.0 (GraphPad Software, La Jolla, CA, USA). At least a 25% reduction (Δ = 250 Ω) in in vitro endothelial resistance was expected following exposure to post-CPB plasma with a standard deviation of 150 Ω [3]. With a significance level (*α*) of 0.05 and beta of 0.9 group sizes of *n* = 8 were calculated. Data are presented as mean ± standard deviation (SD). Changes in endothelial resistance over time were evaluated using repeated measures ANOVA with Bonferroni post-hoc analyses. Two-sided paired t-test were used to evaluate differences between time points. Correlations between circulating angiopoietin-2 levels, endothelial barrier, and microcirculatory perfusion were analyzed using a Pearson correlation test. A *P* value of < 0.05 was considered statistically significant.

## Results

### Patient characteristics

A total of 17 cardiac surgery patients were included in the study. Patient characteristics are listed in Table 1. Patients had a mean age of 67 ± 7 years and were exposed to CPB for 103 ± 18 min with a mean surgical time of 239 ± 38 min. Two patients developed de novo atrial fibrillation, and one patient developed postoperative pulmonary embolisms. No patient developed acute kidney injury, required repeat surgery, or died within 30 days after surgery.

**Table 1:** Patient characteristics and intraoperative and postoperative details

Characteristic	Value
Age (years)	67 ± 7
Male sex (%)	15/17 (88)
Body mass index (kg/m^2^)	29 ± 4
Diabetes mellitus II (%)	2/17 (12)
Hypertension (%)	5/17 (29)
Preoperative lactate (mmol/l)	87 ± 20
Preoperative hemoglobin (mmol/l)	8.4 ± 0.9
Intraoperative details	
Surgery time (min)	239 ± 38
Cardiopulmonary bypass time (min)	103 ± 18
Aortic cross-clamp time (min)	70 ± 14
Anastomoses (*n*)	3 (2–4)
Hemoglobin after onset of CPB (mmol/l)	5.5 ± 0.6
Packed red blood cell transfusion (%) 2 / 17 (12)	12
Fresh frozen plasma transfusion (%) 0 / 17 (0)	0
Thrombocytes (5-donor concentrate; %) 3 / 17 (18)	18
Cell saver transfusion (ml)	491 ± 119
Postoperative details	
Lactate after 24 h (mmol/l)	1.9 ± 0.9*
Hemoglobin after 72 h (mmol/l)	7.0 ± 1.0*
Intensive care length of stay (days)	1 (1–1)
Atrial fibrillation (%)	2 / 17 (12)
Pulmonary embolisms (%)	1 / 17 (6)

Values represent frequencies, means ± standard deviation, or median with interquartile range

*P < 0.05 versus before cardiopulmonary bypass

### Cardiopulmonary bypass-induced renal and pulmonary endothelial hyperpermeability persist in the first postoperative days

**Fig. 1:**
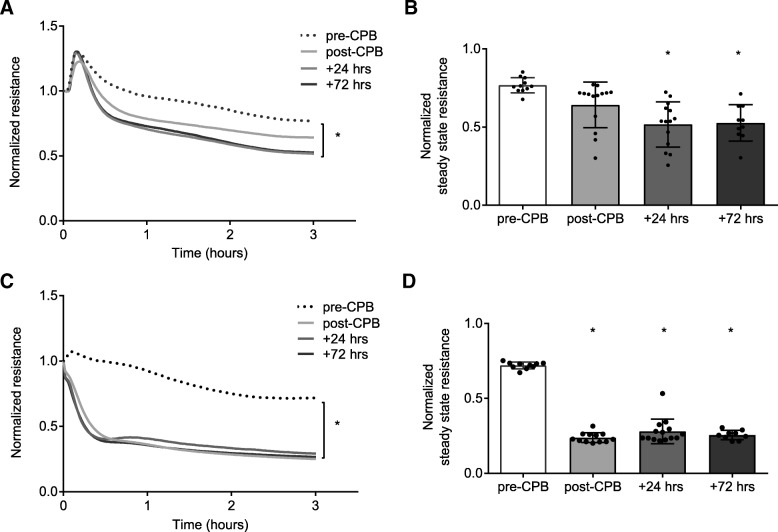
Prolonged postoperative impairment of renal and pulmonary endothelial barrier. Human renal and pulmonary microvascular endothelial cells were exposed to plasma from patients undergoing cardiopulmonary bypass collected before onset of CPB (pre-CPB), after weaning from CPB (post-CPB), and 24 h (+ 24 h) and 72 h (+ 72 h) after surgery. Renal (**a**) and pulmonary (**c**) endothelial resistance after plasma exposure over time and quantification of renal (**b**) and pulmonary (**d**) endothelial resistance after 3 h. Data represent mean or mean ± SD. One-way ANOVA with Bonferroni post-hoc analysis, *P < 0.05 versus pre-CPB; and repeated measures ANOVA, ^#^*P* < 0.05. CPB, cardiopulmonary bypass; SD, standard deviation

Plasma obtained immediately after weaning from CPB reduced renal endothelial barrier function by 17% compared to plasma that was obtained from these patients before CPB (Fig. 1a, b). This reduction in renal endothelial barrier function was even stronger following plasma exposure obtained at 24 h and 72 h after surgery (0.52 ± 0.14 vs. 0.77 ± 0.04, *P* < 0.001 and 0.52 ± 0.11 vs. 0.77 ± 0.04, *P* < 0.001; Fig. 1a, b). In pulmonary endothelial cells, a more severe reduction in endothelial barrier function of 34% was evoked following exposure to plasma obtained after weaning from CPB (0.24 ± 0.03 vs. 0.73 ± 0.02, *P* < 0.001 vs. pre-CPB; Fig. 1c, d). This plasma-induced reduction of pulmonary endothelial barrier function was observed in all samples taken within the first three postoperative days (*P* < 0.001 for all time points vs. pre-CPB; Fig. 1c, d).

### Cardiopulmonary bypass induces in vitro renal and pulmonary intercellular gap formation

**Fig. 2:**
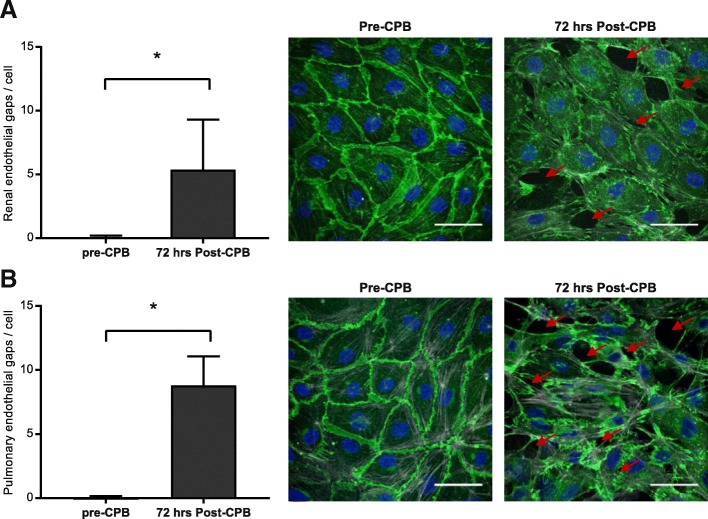
Post-CPB plasma induces renal and pulmonary endothelial gap formation. Quantification of renal (**a**) and pulmonary (**b**) intercellular gap formation and representative images of endothelial cells after exposure of plasma from patients before CPB (pre-CPB, middle panels) and 72 h after CPB (72 h post-CPB, right panels). Endothelial cells were stained for VE-cadherin (adherens junctions; green), actin (stress fibers; white), and DAPI (nuclei; blue) after 3 h of plasma exposure. Red arrows indicate examples of endothelial gaps. Scale bar represents 50 μm. Data represent mean number of gaps per endothelial cell ± SD quantified from *n* = 5 images per time point from 6 patients. One-way ANOVA with Bonferroni post-hoc analysis, *P < 0.05 versus pre-CPB. CPB, cardiopulmonary bypass; SD, standard deviation

Plasma obtained 72 h after CPB increased actin stress fiber formation in renal (4.7 × 10^6^ ± 1.9 × 10^6^ vs. 3.3 × 10^6^ ± 1.6 × 10^6^ integrated fluorescence density per cell, *P* = 0.0016, Additional file 1: Figure S1A, B) and pulmonary endothelial cells (3.7 × 10^6^ ± 2.9 × 10^6^ vs. 2.1 × 10^6^ ± 1.5 × 10^6^ integrated fluorescence density per cell, *P* = 0.03, Additional file 1: Figure S2 A, B) compared to plasma obtained from these patients before CPB. In addition, exposure to plasma obtained 72 h after CPB reduced VE-cadherin at cell-cell contacts in renal (Additional file 1: Figure S1 A, B) and pulmonary (Additional file 1: Figure S2 A, B) endothelial cells. Loss of junctional VE-cadherin after CPB was paralleled by increased renal and pulmonary intercellular gap formation (5.4 ± 3.9 vs. 0.1 ± 0.1 gaps per endothelial cell, *P* < 0.001, Fig. 2a, and 7.9 ± 2.2 vs. 0.1 ± 0.1 gaps per endothelial cell, *P* < 0.001, Fig. 2b, respectively).

### Cardiopulmonary bypass is associated with prolonged postoperative increased angiopoietin-2 levels

**Fig. 3:**
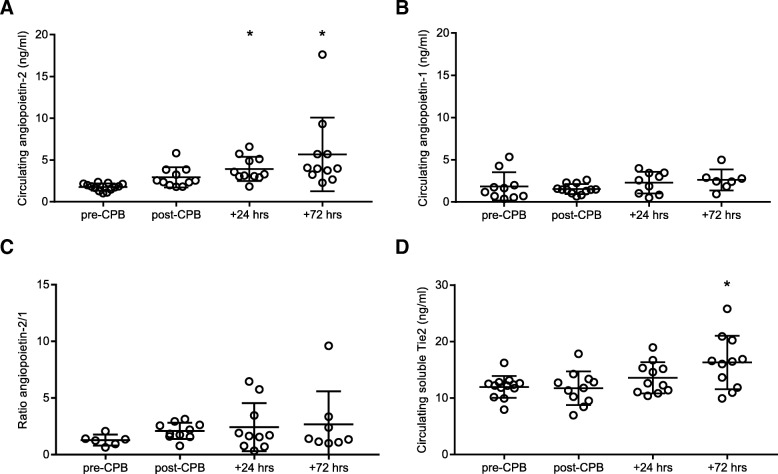
Changes in circulating angiopoietin and soluble Tie2 levels after cardiopulmonary bypass. Circulating levels of angiopoietin-1 (**a**), angiopoietin-2 (**b**), ratio angiopoietin-2/1 (**c**), and soluble Tie2 (**d**) before onset of CPB (pre-CPB), after weaning from CPB (post-CPB), 24 h (+ 24 h) and 72 h (+ 72 h) after surgery corrected for hematocrit levels. Data represent mean + SD. One-way ANOVA with Bonferroni post-hoc analysis, *P < 0.05 versus pre-CPB. CPB, cardiopulmonary bypass; SD, standard deviation

CPB was associated with increased circulating levels of angiopoietin-2 within 24 h after surgery (4.0 ± 1.4 vs. 1.7 ± 0.4 ng/ml, *P* < 0.0001 vs. pre-CPB). Circulating angiopoietin-2 levels further increased in the following 72 postoperative hours (5.7 ± 4.4 vs. 1.7 ± 0.4 ng/ml, *P* < 0.0001 vs. pre-CPB; Fig. 3a). In contrast, circulating angiopoietin-1 levels remained stable in the first 72 h after surgery (2.6 ± 1.2 vs. 1.9 ± 1.7 ng/ml, *P* > 0.9 vs. pre-CPB; Fig. 3b). A twofold rise in angiopoietin-2/1 ratio was found 72 h after surgery compared to pre-CPB (2.8 ± 2.5 vs. 1.2 ± 0.4, *P* = 0.48; Fig. 3c). Circulating levels of the soluble form of the endothelial Tie2 receptor increased 72 h after surgery compared to pre-CPB (16.3 ± 4.7 vs. 11.9 ± 1.9 ng/ml, *P* = 0.018; Fig. 3d).

### Reduced in vitro renal and pulmonary endothelial barrier function are associated with increased angiopoietin-2 levels

**Fig. 4:**
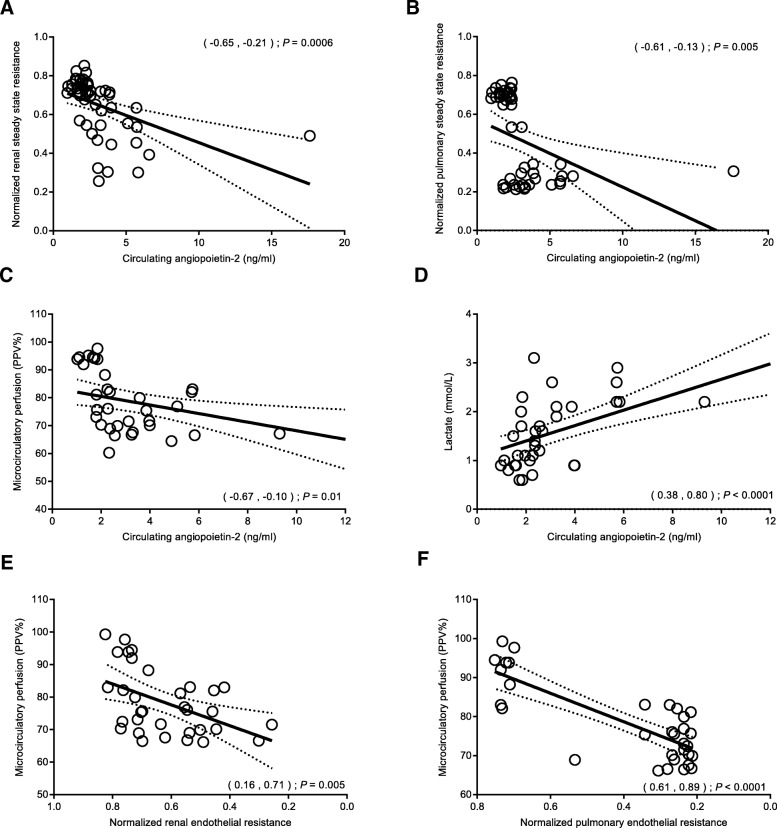
Reduced in vitro renal and pulmonary endothelial barrier are associated with reduced in vivo microcirculatory perfusion and increased angiopoietin-2 levels. Association between circulating angiopoietin-2 levels and renal (**a**) and pulmonary (**b**) endothelial barrier after plasma exposure, microcirculatory perfusion (**c**), and lactate levels (**d**). Association between renal (**e**) and pulmonary (**f**) endothelial barrier function after plasma exposure and microcirculatory perfusion. Data are presented with a linear regression with 95% CI and tested with a Pearson’s correlation test. CPB, cardiopulmonary bypass; CI, confidence interval

Associations were found between plasma-induced reduction in endothelial barrier function and increased angiopoietin-2 levels at corresponding time points in patients undergoing CPB (Fig. 4a, b). During the entire study period, increased circulating levels of angiopoietin-2 correlated with plasma-induced reduction in renal and pulmonary endothelial barrier function (*r* = − 0.46, *P* = 0.0006, Fig. 4a; and *r* = − 0.40, *P* = 0.005, Fig. 4b, respectively).

### Increased angiopoietin-2 levels are associated with postoperative microcirculatory perfusion disturbances

Increased postoperative circulating angiopoietin-2 levels in patients after CPB correlated to reduced microcirculatory perfusion in these patients, as represented by the proportion of perfused vessels (*r* = − 0.43, *P* = 0.01, Fig. 4c). In parallel, plasma lactate, a surrogate marker for impaired tissue perfusion, was positively associated with increased angiopoietin-2 levels (*r* = 0.63, *P* < 0.0001; Fig. 4d). Moreover, plasma-induced reduction in renal and pulmonary endothelial barrier function was associated with reduced microcirculatory perfusion at corresponding time points in these patients (*r* = 0.47, *P* = 0.005, and *r* = 0.79, *P* < 0.001, respectively; Fig. 4e, f).

## Discussion

In this study, we show that cardiac surgery with cardiopulmonary bypass (CPB) is associated with a cell-type specific in vitro endothelial hyperpermeability induced by patient plasma. This plasma-induced renal and pulmonary endothelial hyperpermeability persists until at least 72 h after surgery and were associated with increased circulating angiopoietin-2 levels. In addition, endothelial hyperpermeability as well as increased circulating angiopoietin-2 levels following CPB correlated with in vivo microcirculatory perfusion disturbances. These results suggest that postoperative endothelial hyperpermeability may contribute to delayed recovery of CPB-induced microcirculatory perfusion disturbances, possibly sustained by postoperative release of angiopoietin-2.

Endothelial hyperpermeability is increasingly recognized as a key pathophysiological contributor to postoperative organ dysfunction following cardiac surgery with CPB [7–10]. However, this parameter is clinically limited to the evaluation of fluid overload reflected in pulmonary and renal performance. Despite the growing number of clinical studies evaluating the course of circulating endothelial injury markers, evidence for a causal relation between endothelial barrier dysfunction and impaired oxygenation is scarce and mainly restricted to experimental models [4, 5]. In line with previous studies, we found that the effects of patient plasma withdrawn following CPB associated with reduced in vitro endothelial barrier function. In addition, our results extend previous findings by revealing that this induced loss of endothelial barrier function can be observed in both renal and pulmonary endothelial cells and persists in the first three postoperative days.

Vascular endothelial permeability following CPB-associated systemic inflammation is regulated by several mechanisms of which the angiopoietin/Tie2 system is postulated as central regulator [7–9]. Tie2 is an endothelium-specific transmembrane tyrosine kinase receptor, with angiopoietin-1 and angiopoietin-2 as most dominant ligands [12]. The paracrine agonist angiopoietin-1 protects endothelial integrity by strengthening intercellular junctions. In contrast, the competitive antagonist angiopoietin-2 is released from Weibel-Palade bodies during inflammation and increases endothelial permeability. The observed endothelial barrier disruptive effect of plasma obtained after CPB was more severe in pulmonary endothelium compared to renal endothelium. This could be due to differences in endothelial Tie2-receptor expression levels, since Tie2 is most abundantly expressed in pulmonary microvasculature [21]. Inhibition of Tie2 via angiopoietin-2 following CPB triggers endothelial hyperpermeability by reducing junctional VE-cadherin [22], the essential component of cell-cell junctions. We indeed found that functional loss of endothelial barrier was paralleled with profound changes in cell structures, such as reduced VE-cadherin at cell-cell junctions, increased stress fiber formation, and intercellular gap formation. As both renal and pulmonary function highly depend on intact microvascular barrier, it may not be surprising that complications after CPB mainly present themselves in these organs [12, 13, 16, 23].

Besides its regulatory role in endothelial barrier function, angiopoietin-2 has emerged as a potential early prognostic biomarker [14, 15]. Increased circulating angiopoietin-2 has strongly been linked to the duration of mechanical ventilation, ICU length of stay, positive fluid balance, and increased postoperative organ dysfunction following CPB [10, 16, 23]. In this study, we observed that increased circulating angiopoietin-2 correlated not only with the endothelial barrier disruptive effect of plasma after CPB but also with in vivo microcirculatory perfusion disturbances and lactate levels. Interestingly however, the timing and trend of alterations in angiopoietin-2 do not mirror alterations in microcirculatory perfusion and lactate levels. The delayed increase in angiopoietin-2 following CPB implies that angiopoietin-mediated endothelial barrier dysfunction happens secondary to early CPB-associated endothelial dysfunction and microvascular alterations. These results may suggest that angiopoietin-2 could be involved in prolonging the postoperative leakiness of the endothelium and thereby may attenuate restoration of microcirculatory perfusion following CPB rather than acting as a central mediator during the onset of CPB. In view of these divergent trends, it should also be considered that the found association between angiopoietin-2, endothelial hyperpermeability, and microcirculatory perfusion do not fit with a causal mechanism and may simply reflect a common problem, namely CPB-associated endothelial injury.

Ideally, one would like to further investigate this role of angiopoietin-2 in endothelial permeability and microcirculatory perfusion disturbances by targeting plasma angiopoietin-2 with an antibody blocking its effect leading to attenuation of our described plasma-induced hyperpermeability. Previously, this effect of angiopoietin-2 on endothelial hyperpermeability has been studied in the context of sepsis. Increased endothelial paracellular gap formation induced by serum obtained from sepsis patients could be fully neutralized by blocking angiopoietin-2 [24]. In addition, administration of an angiopoietin-2 inhibitor was found to protect endothelial integrity, reduce pulmonary vascular leakage, and improve survival in experimental sepsis models [25, 26]. Unfortunately, these types of angiopoietin-2 inhibitors are no longer available for experimental testing, and therefore, our results should be interpreted with caution.

Remarkably, all patients in this study showed postoperative increases in angiopoietin-2 levels and plasma-induced in vitro endothelial permeability following CPB. Therefore, the next step would be to identify whether increased angiopoietin-2 may aid in identifying the patients at risk for developing complications and who may possibly benefit from therapy targeted at attenuating postoperative endothelial permeability. When interpreting our data, it is important to mention that we investigated a relatively low-risk cardiac surgery population who were rapidly discharged from the ICU. Studies investigating these alterations in high-risk cardiac surgery populations, who may possibly experience more pronounced alterations in angiopoietin-2 and microcirculatory perfusion, would be of interest to further clarify the clinical implication of our findings.

Besides angiopoietin-2, additional barrier disruptive mediators are involved in CPB-associated endothelial hyperpermeability. Like angiopoietin-2, von Willebrand Factor is stored in Weibel-Palade Bodies and immediately released upon onset of CPB. Release of von Willebrand Factor and generation of thrombin activates coagulation, increases endothelial permeability, and stimulates release of angiopoietin-2. Besides Tie2 inhibition, activation of vascular endothelial growth factor receptor-2 (VEGFR2) is known to increase permeability by internalizing junctional VE-cadherins [27]. Moreover, VEGF is thought to increase permeability by promoting proteolytic cleavage and shedding of the Tie2 receptor [28]. We indeed found increased soluble Tie2 levels at the third postoperative day, suggestive of Tie2 receptor cleavage and shedding after CPB. Altogether, multiple regulatory systems are involved in CPB-associated endothelial hyperpermeability, but all potentiate angiopoietin-2 release. Inhibition of circulating angiopoietin-2 or stimulation of Tie2 activity may therefore provide interesting future therapeutic targets to attenuate postoperative evolution of CPB-associated endothelial hyperpermeability [29–31].

## Conclusions

We showed that cardiac surgery with cardiopulmonary bypass (CPB) associated with a cell-type specific in vitro endothelial hyperpermeability induced by patient plasma. This plasma-induced renal and pulmonary endothelial hyperpermeability persisted until at least 72 h after surgery and corresponded to increased circulating angiopoietin-2 levels. These effects were associated with in vivo microcirculatory perfusion disturbances in corresponding patients. These results suggest that angiopoietin-2 is a biomarker for endothelial hyperpermeability which may contribute to delayed recovery of postoperative microcirculatory perfusion disturbances and the development of organ dysfunction following cardiac surgery with CPB. Whether alterations in angiopoietin-2 may help to identify patients at risk of developing complications, and who may possibly benefit from additional therapy, remains to be investigated in future studies.

## Additional file

Additional file 1: Supplemental methods. **Figure S1.** Changes in renal endothelial cell structures following post-CPB plasma exposure. **Figure S2.** Changes in pulmonary endothelial cell structures following post-CPB plasma exposure. (ZIP 280 kb)

## Abbreviations

ACT: Activated clotting time
ANOVA: Analysis of variance
bM199: Bare M199 medium
CABG: Coronary artery bypass grafting
CI: Confidence interval
cM199: Complete M199 medium
CO_2_: Carbon dioxide
CPB: Cardiopulmonary bypass
DAPI: 4′,6-Diamidino-2-phenylindole
ECIS: Electric cell-substrate impedance sensing
EGF: Endothelial growth factor
eGFR: Estimated glomerular filtration rate
HSA: Human serum albumin
ICU: Intensive care unit
IQR: Interquartile range
PMVEC: Pulmonary microvascular endothelial cell
PPV: Proportion of perfused microvessels
SD: Standard deviation
Tie2: Tyrosine kinase with immunoglobulin-like and EGF-like domains 2
VE-cadherin: Vascular endothelial cadherin
VEGF: Vascular endothelial growth factor
VEGFR2: Vascular endothelial growth factor receptor-2
