# Supplementary material for: Microcirculatory perfusion disturbances following cardiac surgery with cardiopulmonary bypass are associated with in vitro endothelial hyperpermeability and increased angiopoietin-2 levels
Source: Crit Care. 2019 Apr 11;23:117. doi: 10.1186/s13054-019-2418-5 (PMC6460737; doi:10.1186/s13054-019-2418-5)
Supplement: Supplementary file 1 — Supplemental methods. Figure S1. Changes in renal endothelial cell structures following post-CPB plasma exposure. Figure S2. Changes in pulmonary endothelial cell structures following post-CPB plasma exposure. (ZIP 280 kb) [file 13054_2019_2418_MOESM1_ESM.zip › Additional file 1.docx]

**Cell Culture**

Human primary glomerular endothelial cells isolated from human glomerular tissue were obtained from three healthy donors obtained from Cell biologics (H-6014G, Cell Biologics Company, Chicago, United States) and pooled and cultured on gelatin-coated T25 flasks in complete medium in an atmosphere of 95% air and 5% CO_2_ at 37°C. Human pulmonary microvascular endothelial cells were isolated from healthy lung tissue obtained from three donors during lobectomy (Amsterdam UMC – location VU University Medical Center, Amsterdam, The Netherlands) and cultured as described previously [18]. Bare medium (bM199) consisted of Medium 199 supplemented with penicillin 100 U/ml and streptomycin 100 mg/ml (Biowhittaker, Verviers, Belgium). Complete medium for renal endothelial cells consisted of basal medium H1168 supplemented with endothelial cell medium supplemental kit (1168-Kit, Cellbiologics Company, Chicago, United States) containing fetal bovine serum, endothelial cell supplement, L-glutamine, antibiotic and antimycotic supplement, fibroblast growth factor, hydrocortisone, epidermal growth factor, heparin and vascular endothelial growth factor. Complete medium for pulmonary microvascular endothelial cells consisted of basal endothelial cell medium supplemented with 1% endothelial cell growth supplement, 5% fetal calf serum and 1% penicillin streptomycin (ScienCell, Carlsbad, California, United States).

**Endothelial barrier function**

Electric Cell-substrate Impedance Sensing (ECIS, Applied BioPhysics, Troy, New York, United States) was used to measure impedance of endothelial cells. Confluent, passage 4 glomerular endothelial cells or pulmonary microvascular endothelial cells were transferred to gelatin-coated 96-well ECIS culture plates (96W10idf, Applied BioPhysics) that were pre-treated with 10 mM L-cysteine (Merck, Darmstadt, Germany). Approximately 72 hours after transfer, confluent monolayers were confirmed by steady state resistance using continuous ECIS measurements. Confluent monolayers were washed and incubated for one hour with 1% human serum albumin (HSA) in bare medium followed by the addition of 10% platelet-free plasma obtained from cardiac surgery patients at different time points before and after CPB as described above. Resistance of endothelial monolayers was continuously measured at 4.000 Hz for three hours until steady state was reached using ECIS software (v1.2.210.0 PC; Applied Bio-Physics). Measurements were performed in duplicate and data were normalized to baseline.

**Immunofluorescence staining**

Immunofluorescence was used to visualize endothelial cell structures after exposure to plasma obtained from patients either before (*n* = 6) or after (*n* = 6) exposure to CPB. Passage 4 glomerular endothelial cells and pulmonary microvascular endothelial cells were transferred to 5 uM fibronectin-coated coverslips (12 mm x 1,5 mm, Menzel Glazer, Thermo Scientific, Germany) in 24-well culture plates. When confluent monolayers were reached, cells were washed and incubated for one hour with 1% HSA in bare medium followed by the addition of 10% platelet-free plasma. Three hours after plasma exposure, the cells were fixed with 4% paraformaldehyde solution in PBS at room temperature, washed, and permeabilized with 0.2% TritonX-100. Subsequently, coverslips were incubated with primary antibodies against VE-cadherin (SC-6458, 1:400, Santa Cruz, Dallas, Texas, United States) for one hour at room temperature, washed, and incubated with secondary antibody Alexa Fluor 488 (Invitrogen, Carlsbad, California, United States) for one hour at room temperature protected from light. Nuclei and actin were stained using DAPI (1:500; Thermo Fisher Scientific, Waltham, Massachusetts, United States) and acti-stain Phalloidin670 (1:200; donkey anti-goat, Cytoskeleton, Denver, Colorado, United States) respectively. Coverslips were washed and mounted with mowiol on glass slides and stored overnight in the dark at room temperature before images where obtained. Imaging was performed with a NIKON A1R high definition resonant scanning confocal microscope (NIKON Instruments, Amsterdam, The Netherlands) using a 63x Zeiss oil objective. VE-cadherin and actin raw integrated density profiles corrected for total cell fluorescence per image and the number of gaps per nucleus in a minimum of 5 images per time point per patient were quantified using ImageJ.

**Supplemental figure legends**

**Supplemental Figure 1.** Changes in renal endothelial cell structures following post-CPB plasma exposure

Immunofluorescence staining (**A**) of renal endothelial cells after exposure of plasma from patients before CPB (pre-CPB) and 72 hours after CPB (72 hrs post-CPB). Endothelial cells were stained for VE-cadherin (adherens junctions; green), actin (stress fibers; white), and DAPI (nuclei; blue) after three hours of plasma exposure. Quantification (**B**) of actin and of VE-cadherin density in renal endothelial cells after plasma exposure. Red arrows indicate examples of endothelial gaps. Scale bar represents 50 μm. Data represent mean raw integrated density values of corrected total cell fluorescence ± SD quantified from *n* = 5 images per time point from 6 patients. One-way ANOVA with Bonferroni post-hoc analysis, * *P* < 0.05 versus pre-CPB. CPB, cardiopulmonary bypass; SD, standard deviation.

**Supplemental Figure 2.** Changes in pulmonary endothelial cell structures following post-CPB plasma exposure

Immunofluorescence staining (**A**) of pulmonary endothelial cells after exposure of plasma from patients before CPB (pre-CPB) and 72 hours after CPB (72 hrs post-CPB). Endothelial cells were stained for VE-cadherin (adherens junctions; green), actin (stress fibers; white), and DAPI (nuclei; blue) after three hours of plasma exposure. Quantification (**B**) of actin and of VE-cadherin density in pulmonary endothelial cells after plasma exposure. Red arrows indicate examples of endothelial gaps. Scale bar represents 50 μm. Data represent mean raw integrated density values of corrected total cell fluorescence ± SD quantified from *n* = 5 images per time point from 6 patients. One-way ANOVA with Bonferroni post-hoc analysis, * *P* < 0.05 versus pre-CPB. CPB, cardiopulmonary bypass; SD, standard deviation.
